# Optical coherence tomography- vs angiography-guided coronary stent implantation in calcified lesions: the ILUMIEN IV trial

**DOI:** 10.1093/eurheartj/ehaf331

**Published:** 2025-06-05

**Authors:** Ziad A Ali, Doosup Shin, Rajesh Vijayvergiya, Atit A Gawalkar, Richard A Shlofmitz, Fernando Alfonso, Giuseppe Calligaris, Paolo Canova, Koshiro Sakai, Matthew J Price, David Leistner, Francesco Prati, Gary Mintz, Mitsuaki Matsumura, Robert J McGreevy, Robert W McNutt, Hong Nie, Jana Buccola, Ulf Landmesser, Akiko Maehara, Gregg W Stone

**Affiliations:** Department of Cardiology, St. Francis Hospital and Heart Center, Roslyn, NY, USA; New York Institute of Technology, Old Westbury, NY, USA; Department of Cardiology, St. Francis Hospital and Heart Center, Roslyn, NY, USA; Department of Cardiology, Post Graduate Institute of Medical Education and Research, Chandigarh, India; Department of Cardiology, Post Graduate Institute of Medical Education and Research, Chandigarh, India; Department of Cardiology, St. Francis Hospital and Heart Center, Roslyn, NY, USA; Cardiology Department, Hospital Universitario de La Princesa, CIBERCV, IIS-IP, Madrid, Spain; Centro Cardiologico Monzino IRCCS, Milano, Italy; Cardiovascular Department, Ospedale Papa Giovanni XXIII, Bergamo, Italy; Department of Cardiology, St. Francis Hospital and Heart Center, Roslyn, NY, USA; Division of Cardiovascular Diseases, Scripps Clinic, La Jolla, CA, USA; Department of Cardiology, Angiology and Intensive Care Medicine, Deutsches Herzzentrum Charité; Charité—Universitätsmedizin Berlin, Berlin, Germany; Department of Medicine, Cardiology, Goethe University Hospital, Frankfurt, Germany and German Center for Cardiovascular Research (DZHK) Partner Site RheinMain, Frankfurt, Germany; Cardiology, Saint Camillus International University of Health Sciences and CLI Foundation, Rome, Italy; Clinical Trial Center, Cardiovascular Research Foundation, New York, NY, USA; Clinical Trial Center, Cardiovascular Research Foundation, New York, NY, USA; Abbott Vascular, Santa Clara, CA, USA; Abbott Vascular, Santa Clara, CA, USA; Abbott Vascular, Santa Clara, CA, USA; Abbott Vascular, Santa Clara, CA, USA; Department of Cardiology, Angiology and Intensive Care Medicine, Deutsches Herzzentrum Charité; Charité—Universitätsmedizin Berlin, Berlin, Germany; Berlin Institute of Health, Berlin, Germany; German Centre for Cardiovascular Research, Berlin, Germany; Clinical Trial Center, Cardiovascular Research Foundation, New York, NY, USA; Center for Interventional Cardiovascular Care, Columbia University Medical Center, New York, NY, USA; The Zena and Michael A. Wiener Cardiovascular Institute, Icahn School of Medicine at Mount Sinai, New York, NY, USA

**Keywords:** Optical coherence tomography, Percutaneous coronary intervention, Stent, Prognosis

## Abstract

**Background and Aims:**

The large-scale, randomized ILUMIEN IV trial was examined to determine whether procedural guidance with optical coherence tomography (OCT) during percutaneous coronary intervention (PCI) of angiographically calcified lesions improves outcomes.

**Methods:**

Patients with a single PCI target lesion were included in the present analysis. The presence of none, mild, moderate or severe lesion calcification was determined by an angiographic core laboratory. The primary imaging endpoint was the post-PCI minimal stent area (MSA) assessed by OCT. The primary clinical endpoint was 2-year target-vessel failure (TVF), a composite of cardiac death, target-vessel myocardial infarction (TV-MI), or ischaemia-driven target-vessel revascularization.

**Results:**

In the overall population (*n* = 2114), there was a significant interaction between the effect of randomization to OCT guidance vs angiography guidance in lesions with moderate/severe calcification (*n* = 1082) vs no/mild calcification (*n* = 1032) on the 2-year rate of TVF (*P*interaction = .01). The post-PCI MSA in moderately and severely calcified lesions was larger with OCT guidance (*n* = 544) compared with angiography guidance (*n* = 538) (5.57 ± 1.86 mm^2^ vs 5.33 ± 1.78 mm^2^; *P* = .03). In the moderate/severe calcified lesion cohort, TVF within 2 years occurred in 35 patients with OCT guidance and in 51 patients with angiography guidance (6.8% vs 9.7%; adjusted hazard ratio [aHR] 0.62; 95% confidence interval [CI] 0.40–0.96), whereas there was no significant difference in TVF in the no/mild calcified lesion cohort (7.7% vs 5.2%; aHR 1.48; 95% CI 0.90–2.44) (*P*interaction = .01). In moderately/severely calcified lesions, OCT-guided PCI also reduced the 2-year rates of serious major adverse cardiac events (2.8% vs 4.7%; aHR 0.49; 95% CI 0.25–0.95; *P* = .03), TV-MI (1.9% vs 4.0%; aHR 0.36; 95% CI 0.17–0.79; *P* = .01), and stent thrombosis (0.2% vs 1.5%; aHR 0.11; 95% CI 0.01–0.89; *P* = .04) compared with angiography-guided PCI.

**Conclusions:**

In the ILUMIEN IV trial, OCT-guided PCI in patients with angiographically determined moderately or severely calcified lesions reduced the 2-year rate of TVF compared with angiography-guided PCI, an effect that was not seen in patients with lesions with no or mild angiographic calcium.

## Structured Graphical Abstract

**Structured Graphical Abstract:**
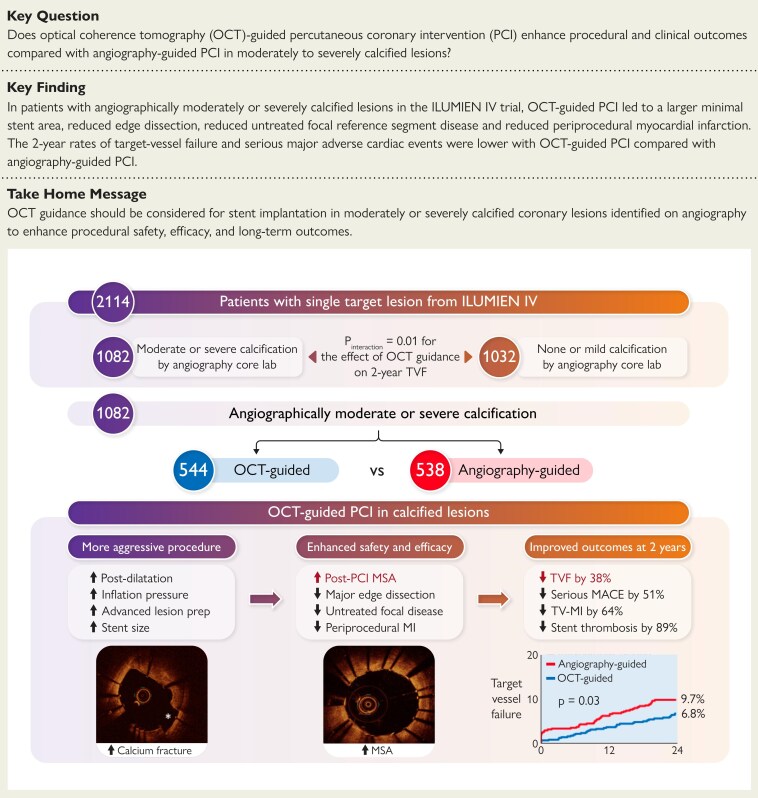

**See the editorial comment for this article ‘Mastering percutaneous coronary intervention in calcified coronary lesions with optical coherence tomography: the path to optimal outcomes’, by M. Belmonte and E. Barbato, https://doi.org/10.1093/eurheartj/ehaf302.**

## Introduction

Despite advancements in technology, coronary artery calcification remains a significant challenge in percutaneous coronary intervention (PCI). PCI of calcified coronary lesions is associated with an increased risk of suboptimal stent implantation, procedural complications, and long-term adverse events.^1–4^ In this regard, identification and appropriate management of calcified coronary lesions are crucial to improve patient outcomes after PCI.

The ability of angiography to identify detailed morphological features of coronary artery calcification is limited, a drawback that can lead to suboptimal stent implantation.^5^ In contrast, intravascular imaging, especially optical coherence tomography (OCT), is able to characterize and quantify coronary artery calcification, facilitating appropriate lesion preparation strategies prior to stent implantation.^4,6^ Furthermore, intravascular imaging allows for the identification and correction of under-expanded stents, which are commonly seen in calcified coronary lesions and have been associated with worse clinical outcomes.^7,8^ These theoretical benefits are supported by recent randomized controlled trials (RCTs) and a meta-analysis demonstrating improved outcomes following intravascular imaging-guided PCI compared with angiography-guided PCI in patients with complex coronary lesions.^9–13^ However, no prior RCT has reported detailed outcomes specifically in patients with calcified coronary lesions undergoing PCI, and it remains unclear whether and how OCT guidance can improve procedural and clinical outcomes in this complex lesion subset.

We therefore aimed to compare OCT-guided PCI with angiography-guided PCI in the subgroup of patients with angiographically calcified coronary lesions in the ILUMIEN IV: OPTIMAL PCI trial.

## Methods

### Trial design and study population

ILUMIEN IV: OPTIMAL PCI was a large-scale, prospective, multicontinental, single-blind randomized trial that compared OCT-guided PCI with angiography-guided PCI in patients with high-risk clinical characteristics and/or complex lesions. Detailed specifics of this trial have been previously documented,^10,14^ and a full list of inclusion and exclusion criteria can be found in the Supplementary data online, Methods. In brief, ILUMIEN IV included patients with medically treated diabetes mellitus and/or culprit lesions responsible for a recent myocardial infarction (MI), long or multiple lesions with intended total stent length ≥28 mm, a bifurcation lesion requiring a two-stent strategy, a severely calcified lesion, a chronic total occlusion (CTO), or diffuse or multi-focal in-stent restenosis (ISR).

From the original trial population, patients undergoing PCI of a single coronary artery lesion in a major epicardial vessel were included in the present analysis. Angiographically moderate or severe calcification was defined by the core laboratory as radio-opacities seen only during the cardiac cycle (moderate) or without cardiac motion generally on both sides of the arterial lumen (severe).^4,15^

The trial adhered to the principles of the Declaration of Helsinki and received approval from the institutional review board at each participating site.^14^ All patients provided written informed consent. The trial was funded by Abbott (Santa Clara, CA, USA) who also participated in data analysis.

### Interventions

Percutaneous coronary intervention was performed using standard techniques with fluoropolymer-coated everolimus-eluting stents (XIENCE, Abbott Vascular, Santa Clara, CA, USA). In the OCT-guided group, a pre-specified protocol was utilized to guide and optimize stent implantation.^14^ In brief, pre-PCI OCT was used to assess plaque morphology, stent size, and proximal and distal reference segments. Stent size was determined based on the mean external elastic lamina (EEL)-based distal reference vessel diameter, rounded down to the nearest stent diameter, or the mean lumen-based vessel diameter, rounded up to the nearest stent diameter if the EEL was not adequately visualized. Following stent implantation, post-PCI OCT was performed, and post-dilatation was conducted to ensure adequate stent expansion (minimal stent area [MSA] ≥90% of the closest reference segment). In cases of major edge dissection or residual focal inflow/outflow disease in the reference segments, additional stenting was recommended. A final OCT run was mandated in both the OCT-guided and angiography-guided groups, with operators in the angiography-guided group being blinded to the final OCT images. All angiography and OCT images were analysed at an independent core laboratory (Cardiovascular Research Foundation, New York, NY, USA). Further details about the procedure and specific study protocol have been previously reported.^10,14^

### Study endpoints

The primary imaging endpoint was the final post-PCI MSA. The primary clinical endpoint was the 2-year rate of target-vessel failure (TVF), a composite of cardiac death, target-vessel MI (TV-MI), or ischemia-driven target-vessel revascularization (ID-TVR). The secondary endpoints included the individual components of TVF, definite or probable stent thrombosis within 2 years, and serious major adverse cardiac events (MACE), a composite of cardiac death, TV-MI, or definite or probable stent thrombosis. As a safety endpoint, serious MACE was also assessed at 30 days.

All deaths were classified as cardiac unless a clear non-cardiac cause was identified. Procedural and spontaneous MIs were defined using the modified Academic Research Consortium (ARC)-2 criteria^16^ and the 4th Universal definition,^17^ respectively. Revascularization was deemed ischemia-driven if it met any of the following criteria: a positive functional study, diameter stenosis ≥50% with anginal symptoms, or ≥70% without angina or a positive functional study. Definite or probable stent thrombosis was defined according to the modified ARC-2 criteria. Detailed definitions are available in the prior publication^10^ and the Supplementary data online, Method. An independent clinical endpoint committee adjudicated all clinical events.

### Statistical analysis

All analyses were conducted on an intention-to-treat basis. Continuous variables were presented as mean ± standard deviation, while categorical variables were presented as numbers and relative frequencies (%). Comparisons were made using a two-sample *t*-test for continuous variables and a χ^2^ test for categorical variables. The incidences of clinical endpoints were presented as Kaplan–Meier estimates. Multivariable Cox proportional hazard regression models were used to estimate adjusted hazard ratios (aHR) and 95% confidence intervals (CI) for the clinical endpoints. Covariates in these models included age, sex, hypertension, hyperlipidaemia, diabetes, prior MI, prior PCI, prior coronary artery bypass graft surgery, and complex lesion features including a bifurcation lesion with intended 2-stent strategy, CTO, long lesion, and ISR. Subgroup analysis was performed according to age (<65 years vs ≥65 years), sex, acute coronary syndrome on presentation, medication-treated diabetes mellitus, lesion complexity (long or multiple lesions vs short or single lesions), bifurcation, culprit lesions of MI, CTO, and ISR. In the overall trial population, the interaction between the severity of angiographic calcification (moderate/severe vs no/mild) and the impact of OCT guidance on TVF was examined by formal interaction testing. All probability values were two-sided, and *P*-values <.05 were considered statistically significant. All statistical analyses were performed using SAS v9.4 (SAS Institute Inc, Cary, NC, USA).

## Results

### Study population and interaction analysis

ILUMIEN IV enrolled 2487 patients from 17 May 2018 to 29 December 2020 at 80 sites in 18 countries. Among patients with a single treated lesion in a major epicardial vessel (*n* = 2114), by core laboratory determination the lesion was angiographically moderately or severely calcified in 1082 patients (51.2%) and had absent or mild calcification in 1032 patients (48.8%) (see Supplementary data online, *Figure S1*). With angiographic guidance alone, TVF at 2 years occurred more frequently after PCI in moderately or severely calcified lesions compared with no or mildly calcified lesions (9.7% vs 5.2%; hazard ratio [HR] 1.93; 95% CI 1.21–3.10; aHR 1.88; 95% CI 1.16–3.04). In contrast, with OCT guidance, the 2-year rates of TVF were similar after PCI in moderately or severely calcified lesions and in no or mildly calcified lesions (6.8% vs 7.7%; HR 0.84; 95% CI 0.53–1.33; aHR 0.66; 95% CI 0.41–1.07). In the moderate/severe calcified lesion cohort, TVF within 2 years occurred in fewer patients with OCT guidance compared with angiography guidance (6.8% vs 9.7%; aHR 0.62; 95% CI 0.40–0.96), whereas there was no significant difference in TVF in the no/mild calcified lesion cohort (7.7% vs 5.2%; aHR 1.48; 95% CI 0.90–2.44) (*P*_interaction_ = 0.01). Hence, we chose to focus specifically on understanding the impact of OCT guidance in angiographically moderately or severely calcified lesions.

### Patient and lesion characteristics

Baseline patient and qualifying complex lesion characteristics were similar between the OCT-guided and angiography-guided groups in patients with angiographically moderately or severely calcified lesions (*Table 1*) and in those with lesions that had no or mild calcification (see Supplementary data online, *Table S1*). Among patients with angiographically moderately or severely calcified lesions, baseline measures and final angiographic outcomes were also well-balanced between the two treatment groups, except for a slightly longer lesion length observed in the OCT-guided group (*Table 2*). Specifically, angiographically severe calcification was present in 60.3% and 59.7% of the lesions in the OCT-guided and angiography-guided groups, respectively (*P* = .83).

**Table 1:** Baseline characteristics of patients with moderate or severe lesion calcification

Characteristic	OCT-guided (*N* = 544)	Angiography-guided (*N* = 538)
Age, year	67.3 ± 10.1	67.0 ± 10.0
Male	420 (77.2%)	410 (76.2%)
BMI, kg/m^2^	28.7 ± 5.4	28.7 ± 5.5
Hypertension	393 (72.2%)	411 (76.4%)
Dyslipidaemia	353 (64.9%)	368 (68.4%)
Current or recent smoker^a^	91 (16.7%)	83/537 (15.5%)
Peripheral vascular disease	26 (4.8%)	37 (6.9%)
Prior myocardial infarction	106 (19.5%)	118 (21.9%)
Prior PCI in the target vessel	71/530 (13.4%)	76/530 (14.3%)
Prior CABG	32 (5.9%)	26 (4.8%)
Renal insufficiency^b^	51 (9.4%)	46 (8.6%)
Dialysis	15 (2.8%)	14 (2.6%)
Left ventricular ejection fraction, %	55.3 ± 8.5	55.5 ± 8.8
Clinical presentation		
Silent ischaemia	82 (15.1%)	89 (16.5%)
Stable angina	157 (28.9%)	174 (32.3%)
Unstable angina	167 (30.7%)	157 (29.2%)
NSTEMI	110 (20.2%)	85 (15.8%)
Recent STEMI (>24 h)	28 (5.1%)	33 (6.1%)
Qualifying characteristics^c^		
Diabetes mellitus, medication-treated	226 (41.5%)	213 (39.6%)
Culprit lesion NSTEMI	110 (20.2%)	85 (15.8%)
Culprit lesion STEMI >24 h	27 (5.0%)	31 (5.8%)
Long or multiple lesions^d^	398 (73.2%)	65 (67.8%)
Two-stent bifurcation^e^	18 (3.3%)	15 (2.8%)
Severe calcification^f^	87 (16.0%)	96 (17.8%)
Chronic total occlusion^g^	31 (5.7%)	27 (5.0%)
Diffuse or multi-focal ISR	61 (11.2%)	62 (11.5%)

Data are presented as means ± standard deviation or number (percentage).

CABG, coronary artery bypass graft; ISR, in-stent restenosis; NSTEMI, non-ST-segment elevation myocardial infarction; PCI, percutaneous coronary intervention; STEMI, ST-segment elevation myocardial infarction.

^a^Recent smoking within 30 days.

^b^Estimated glomerular filtration rate <60 mL/min.

^c^Patients may have more than one qualifying characteristic.

^d^Total stent length ≥28 mm.

^e^Intended for treatment with a stent ≥2.5 mm in diameter in both the main vessel and side-branch vessel.

^f^Defined as visible calcification in the absence of cardiac motion, generally on both sides of the vessel wall.

^g^After successful crossing with antegrade wire escalation and pre-dilatation.

**Table 2:** Angiographic characteristics (core-laboratory assessed) of lesions with moderate or severe calcification

Characteristic	OCT guided	Angiography guided
Pre-PCI	*L* = 544^a^	*L* = 538^a^
Target vessel		
Left anterior descending	317 (58.3%)	312 (58.0%)
Left circumflex	82 (15.1%)	70 (13.0%)
Right coronary artery	145 (26.7%)	156 (29.0%)
Thrombus	30 (5.5%)	28/537 (5.2%)
Severe calcification	328 (60.3%)	321 (59.7%)
Moderate calcification	216 (39.7%)	217 (40.3%)
Reference vessel diameter, mm	2.94 ± 0.41	2.94 ± 0.40
Minimum lumen diameter, mm	0.88 ± 0.41	0.91 ± 0.42
Diameter stenosis, %	69.7 ± 13.4	68.9 ± 13.5
Lesion length, mm	34.9 ± 15.3	32.6 ± 16.7
Baseline TIMI III flow^b^	454/543 (83.6%)	443 (82.3%)
Post-PCI	*L* = 534^a^	*L* = 533^a^
Reference vessel diameter, mm^c^	2.98 ± 0.42	2.96 ± 0.39
Acute lumen gain, mm^c^	1.75 ± 0.51	1.70 ± 0.51
Minimum lumen diameter, mm^c^	2.64 ± 0.41	2.62 ± 0.39
Diameter stenosis, %^c^	11.4 ± 6.3	11.4 ± 6.4
Final TIMI III flow^b^	522/532 (98.1%)	521 (97.7%)

Data are presented as means ± standard deviation or number (percentage).

OCT, optical coherence tomography; PCI, percutaneous coronary intervention; TIMI, Thrombolysis in Myocardial Infarction.

^a^Total number of main vessel lesions from the angiography core laboratory. Analysis is based on available data.

^b^Per vessel analysis.

^c^In-stent analysis.

In the OCT-guided group, pre-PCI OCT in patients with angiographically moderately or severely calcified lesions demonstrated a minimal lumen area of 1.95 ± 0.81 mm, maximum superficial calcium arc of 205.1 ± 99.5° and maximum superficial calcium thickness of 0.82 ± 0.27 mm (see Supplementary data online, *Table S2*).

### Procedural characteristics

OCT guidance in moderately or severely calcified lesions resulted in use of longer total stent length and greater procedure duration, fluoroscopy time, total radiation dose, and contrast volume compared with angiography guidance (*Table 3*). Additionally, OCT guidance led to more frequent advanced lesion preparation with a cutting or scoring balloon, atherectomy, lithotripsy, or laser, larger maximum stent diameters, larger maximum device sizes, and more frequent post-dilatation with a greater number of balloons and higher inflation pressures compared with angiography guidance (*Table 3*).

**Table 3:** Procedural characteristics in patients with moderate or severe lesion calcification

Characteristic	OCT-guided	Angiography-guided	*P*-value
Procedural characteristics, per patient	*N* = 544	*N* = 538	
Radial access	370 (68.0%)	375 (69.7%)	.55
Total stent length, mm	43.7 ± 20.4	39.8 ± 21.2	.002
Procedure duration, min	67.7 ± 38.0	51.5 ± 37.8	<.001
Fluoroscopy duration, min	21.3 ± 14.2	18.0 ± 12.7	<.001
Total radiation dose, Gy	2.02 ± 1.77	1.64 ± 1.56	.001
Contrast volume, mL	230.4 ± 88.6	196.5 ± 85.2	<.001
Procedural characteristics, per lesion	L = 553^a^	L = 546^a^	
Advanced lesion preparation performed^b^	97 (17.5%)	54 (9.9%)	<.001
Maximum stent diameter, mm	3.22 ± 0.46	3.16 ± 0.38	.02
Maximum device size, mm^c^	3.70 ± 0.55	3.42 ± 0.46	<.001
Post-dilatation performed	539 (97.5%)	473 (86.6%)	<.001
Number of post-dilatation balloons used	1.8 ± 1.2	1.5 ± 1.3	<.001
Maximum inflation pressure, atm	20.0 ± 3.1 (552)	18.6 ± 3.3	<.001

Data are presented as means ± standard deviation or number (percentage).

Atm, atmospheres; Gy, Gray; ISR, in-stent restenosis; NSTEMI, non-ST-segment elevation myocardial infarction; and STEMI, ST-segment elevation myocardial infarction.

^a^Total number of treated main vessel target lesions reported by participating sites. Analysis is based on available data and excludes missing subjects/lesions for whom the data field was not completed or the case report form was missing.

^b^Advanced lesion preparation includes pre-stent treatment with a cutting or scoring balloon, atherectomy, lithotripsy, or laser.

^c^The maximum device size and the maximum inflation pressure represent either the stent size and maximum inflation pressure or the post-dilatation balloon and maximum inflation pressure, whichever is the larger of the two.

### Imaging endpoints

OCT-guided PCI in moderately or severely calcified lesions led to a larger final MSA compared with angiography-guided PCI (5.57 ± 1.86 mm^2^ vs 5.33 ± 1.78 mm^2^; difference: 0.24 mm^2^, 95% CI 0.02–0.45; *P* = .03) (*Table 4*). In addition, OCT-guided PCI was associated with significantly greater minimal and mean stent expansion compared with angiography-guided PCI. While the frequency of complete calcium fracture (a new disruption through the full thickness of a calcified plaque; Supplementary data online, *Figure S2A*) was similar in both groups, partial calcium fracture (a new disruption that does not extend through the entire thickness of the calcified plaque; Supplementary data online, *Figure S2B*) was more frequently observed in the OCT-guided group than in the angiography-guided group (19.7% vs 14.3%; *P* = .02). Conversely, any or major edge dissection, any or major malapposition, and untreated focal disease in reference segments were more frequently observed in the angiography-guided group than in the OCT-guided group (*Table 4*).

**Table 4:** Post-PCI OCT results (core laboratory assessed) of lesions with moderate or severe calcification

Characteristic	OCT-guided (*L* = 544)	Angiography-guided (*L* = 538)	Difference (95% CI)	*P*-value
Minimal stent area, mm^2^	5.57 ± 1.86	5.33 ± 1.78	0.24 (0.02, 0.45)	.03
Minimal stent expansion, %	78.7 ± 16.8	75.2 ± 17.4	3.52 (1.48, 5.56)	<.001
Mean stent expansion, %	109.8 ± 16.4	101.8 ± 17.9	8.07 (6.02, 10.12)	<.001
Acceptable expansion ≥90%^a^	186 (34.2%)	108 (20.1%)	14.1% (8.8%, 19.3%)	<.001
Proximal reference lumen area, mm^2^	8.71 ± 3.17	8.60 ± 3.16	0.12 (−0.26, 0.50)	.54
Distal reference lumen area, mm^2^	5.70 ± 2.18	5.91 ± 2.30	−0.21 (−0.47, 0.06)	.13
Calcium fracture,^b^ any	194 (35.7%)	172 (32.0%)	3.7% (−1.9%, 9.3%)	.20
Complete	161 (29.6%)	156 (29.0%)	0.6% (−4.8%, 6.0%)	.83
Partial	107 (19.7%)	77 (14.3%)	5.4% (0.9%, 9.8%)	.02
Edge dissection, any^c^	167/543 (30.8%)	212/537 (39.5%)	−8.7% (−14.3%, −3.0%)	.003
Major^d^	12/543 (2.4%)	25/537 (4.7%)	−2.5% (−4.8%, −0.3%)	.03
Minor	155/543 (28.5%)	187/537 (34.8%)	−6.3% (−11.8%, −0.8%)	.03
Malapposition, any^e^	358 (65.8%)	433 (80.5%)	−14.7% (−19.8%, −9.4%)	<.001
Major	108 (19.9%)	215 (40.0%)	−20.1% (−25.4%, −14.7%)	<.001
Minor	250 (46.0%)	218 (40.5%)	5.4% (−0.5%, 11.3%)	.07
Tissue protrusion, any	295 (54.2%)	240 (44.6%)	9.6% (3.7%, 15.5%)	.002
Major^f^	31 (5.7%)	47 (8.7%)	−3.0% (−6.2%, 0.1%)	.053
Minor	264 (48.5%)	193 (35.9%)	12.7% (6.8%, 18.4%)	<.001
Reference segment disease, any^c^	104/517 (20.1%)	120/517 (23.2%)	−3.1% (−8.1%, 1.9%)	.23
Focal^g^	52/517 (10.1%)	73/517 (14.1%)	−4.1% (−8.1%, −0.1%)	.045
Diffuse	52/517 (10.1%)	47/517 (9.1%)	1.0% [−2.7%, 4.6%]	.60

Data are presented as mean ± standard deviation and number (percentage).

CI, confidence interval; *L*, number of lesions; OCT, optical coherence tomography.

^a^Defined as minimal stent area of the proximal segment ≥90% of the proximal reference lumen area and minimal stent area of the distal segment ≥90% of the distal reference lumen area.

^b^A calcium fracture is identified as a new disruption or break in the continuity of a calcified plaque, which can extend either through the entire thickness of the calcium layer (complete fracture) or only partially through the thickness (partial fracture). A single lesion may include both complete and partial fractures.

^c^Some cases were excluded due to incomplete visualization of segments of the OCT pullback precluding the assessment.

^d^Defined as ≥60° of the circumference of the vessel at site of dissection and ≥3 mm in length.

^e^Malapposition is defined as stent struts clearly separated from the vessel wall without any tissue behind the struts with a distance from the adjacent intima of ≥0.2 mm and not associated with any side branch. Major malapposition is defined as malapposition associated with unacceptable stent expansion.

^f^Protrusion area/stent area at site of tissue protrusion ≥10% and the minimal intrastent flow area (minimal stent area—protrusion area) is unacceptable (<90% of respective proximal or distal reference area.

^g^Refers to untreated reference segment disease, which is defined as focal disease with untreated minimal lumen area <4.5 mm^2^ within 5 mm from the proximal and/or distal stent edges.

### Clinical outcomes

**Figure 1:**
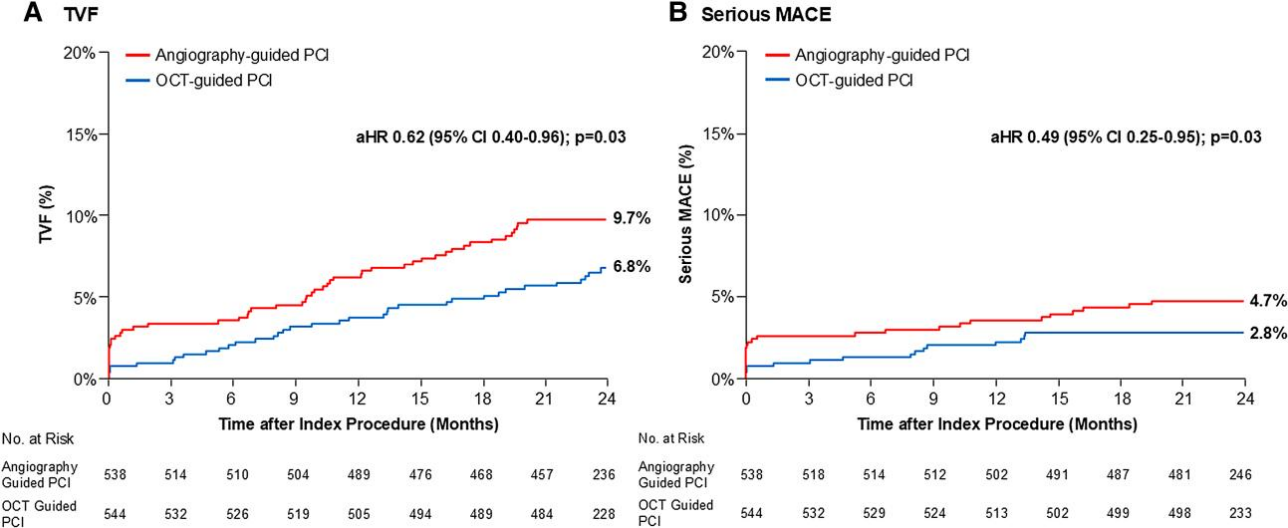
Time-to-first composite outcomes after PCI in angiographically moderately or severely calcified lesions. Kaplan–Meier curves of time to (*A*) TVF (a composite of cardiac death, target vessel-related myocardial infarction, or ischaemia-driven target-vessel revascularization) and (*B*) serious MACE (a composite of cardiac death, target vessel-related myocardial infarction, or definite/probable stent thrombosis) through 2-year follow-up are presented. aHR, adjusted hazard ratio; CI, confidence interval; MACE, major adverse cardiac event; OCT, optical coherence tomography; PCI, percutaneous coronary intervention; TVF, target-vessel failure

Among patients with angiographically moderately or severely calcified lesions, TVF within 2 years occurred in 35 patients after OCT-guided PCI and in 51 patients after angiography-guided PCI (6.8% vs 9.7%; aHR 0.62; 95% CI 0.40–0.96; *P* = .03) (*Table 5* and *Figure 1A*). OCT-guided PCI also resulted in reduced 2-year risks of serious MACE (2.8% vs 4.7%; aHR 0.49; 95% CI 0.25–0.95; *P* = .03) (*Figure 1B*), TV-MI (1.9% vs 4.0%; aHR 0.36; 95% CI 0.17–0.79; *P* = .01), and definite or probable stent thrombosis (0.2% vs 1.5%; aHR 0.11; 95% CI 0.01–0.89; *P* = .04) compared with angiography-guided PCI (*Table 5* and Supplementary data online, *Figure S3*). Additionally, OCT-guided PCI resulted in fewer serious MACE within 30 days (0.7% vs 2.6%; aHR 0.24; 95% CI 0.08–0.74; *P* = .01). The risk of TVF at 2 years was consistent across subgroups (see Supplementary data online, *Figure S4*).

**Table 5:** Clinical outcomes of patients with moderate or severe lesion calcification

Characteristic	OCT-guided (*N* = 544)	Angiography-guided (*N* = 538)	HR (95% CI)	Adjusted HR (95% CI)^a^	*P*-value
Target-vessel failure,^b^ 2-year	35 (6.8%)	51 (9.7%)	0.66 (0.43, 1.02)	0.62 (0.40, 0.96)	.03
Cardiac death	5 (0.9%)	9 (1.7%)	0.55 (0.18, 1.63)	0.52 (0.17, 1.58)	.25
Target-vessel MI	10 (1.9%)	21 (4.0%)	0.47 (0.22, 0.99)	0.36 (0.17, 0.79)	.01
Periprocedural MI	4 (0.7%)	12 (2.2%)	0.33 (0.11, 1.02)	0.29 (0.09, 0.91)	.03
Spontaneous MI	6 (1.1%)	10 (1.9%)	0.59 (0.21, 1.62)	0.43 (0.14, 1.28)	.13
Target-vessel revascularization, ischaemia-driven	28 (5.5%)	35 (6.8%)	0.78 (0.47, 1.28)	0.73 (0.44, 1.22)	.23
Definite or probable stent thrombosis, 2-year	1 (0.2%)	8 (1.5%)	0.12 (0.02, 0.98)	0.11 (0.01, 0.89)	.04
Serious MACE,^c^ 2-year	15 (2.8%)	25 (4.7%)	0.59 (0.31, 1.11)	0.49 (0.25, 0.95)	.03
Safety composite endpoint,^c^ 30-day	4 (0.7%)	14 (2.6%)	0.28 (0.09, 0.86)	0.24 (0.08, 0.74)	.01

Data are presented as number of events (Kaplan–Meier estimated event rates).

CI, confidence interval; HR, hazard ratio; MI, myocardial infarction; OCT, optimal coherence tomography.

^a^Adjusted by age, sex, hypertension, hyperlipidaemia, diabetes, prior MI, prior percutaneous coronary intervention, prior coronary artery bypass graft surgery, and complex lesion features including bifurcation with intended 2-stent strategy, chronic total occlusion, long lesions ≥28 mm, and in-stent restenosis.

^b^The composite of cardiac death, target-vessel MI, or ischaemia driven target-vessel revascularization.

^c^The composite of cardiac death, target-vessel MI, or stent thrombosis.

## Discussion

In this substudy of the ILUMIEN IV trial, we report the following major findings: (i) by core laboratory analysis, angiographically moderately or severely calcified lesions were frequent, comprising 51.2% of all PCI target lesions; (ii) in the overall patient population, a significant interaction was present between the presence of angiographically moderately or severely calcified lesions and the impact of OCT guidance on TVF after PCI. Specifically, the rate of 2-year TVF after PCI was nearly two-fold increased with angiographic guidance in lesions with moderate or severe calcification compared with those with no or mild calcification. Conversely, OCT guidance mitigated this risk so the rates of 2-year TVF were similar regardless of the angiographic severity of calcification—this interaction was significant in unadjusted and adjusted analyses; (iii) in patients with angiographically moderate or severe coronary artery calcification undergoing PCI, OCT guidance led to more frequent use of advanced lesion preparation, greater maximum stent diameter and device size, and more frequent post-dilatation with higher inflation pressures compared with angiography guidance; (iv) OCT-guided PCI resulted in a greater degree of plaque modification, with more frequent partial calcium fractures, and greater final MSA and stent expansion compared with angiography-guided PCI; (v) OCT-guided PCI improved the 30-day safety of the PCI procedure and also reduced the 2-year risks of TVF, serious MACE, TV-MI, and stent thrombosis compared with angiography-guided PCI (*Structured Graphical Abstract*).

Although calcified coronary lesions are stable atherosclerotic plaques with a more favourable natural history compared with lipidic plaques,^5,18^ PCI of calcified lesions is associated with a higher risk of adverse procedural and long-term clinical outcomes than PCI of non-calcified lesions.^1–4^ This is in part due to technical challenges during PCI as reduced vessel compliance from lesion calcification increases the risk of procedural failure, vessel damage and complications, and suboptimal stent implantation.^4^ Intravascular imaging can play a pivotal role in overcoming these challenges. First, intravascular imaging enables the characterization and quantification of coronary artery calcification, which cannot be done by angiography alone. Calcium scoring systems based on intravascular imaging parameters have been developed to predict suboptimal stent implantation, thereby informing the need for advanced lesion preparation.^6,19^ Second, intravascular imaging can help select the appropriate plaque modification tools and device sizes by precisely assessing wire bias and measuring reference vessel size. This not only provides confidence to operators but also maximizes the chance of achieving an optimal MSA, while avoiding complications such as dissection or perforation. Third, post-PCI intravascular imaging can provide crucial information to optimize stent implantation by directing additional balloon inflations to improve stent expansion and additional stent implantation, if necessary, to correct dissections or treat residual reference segment disease, all of which are linked to worse clinical outcomes.^20^ In this context, consensus statements from interventional societies in both Europe and the US recommend planning and optimizing stent implantation for calcified coronary lesions using intravascular imaging guidance.^4,21^

Multiple large-scale RCTs have consistently shown the benefits of intravascular imaging-guided PCI compared with angiography-guided PCI, particularly in patients with high-risk clinical characteristics or complex lesions.^9–11,13^ In a recent substudy from ILUMIEN IV, OCT-guided stent implantation was shown to reduce the risk of serious MACE compared with angiography guidance alone in complex coronary lesions as assessed by the site, including long lesions, bifurcations, severe calcifications, CTO, and ISR.^13^ Although none of these RCTs were specifically designed or powered for patients with calcified coronary lesions, a recent study-level meta-analysis of four large-scale RCTs suggested that intravascular imaging-guided PCI reduces the risk of subsequent MACE compared with angiography-guided PCI in that patient cohort.^12^

Unlike the recent complex lesion substudy of ILUMIEN IV,^13^ the present study specifically evaluated the impact of OCT-guided stent implantation in heavily calcified coronary lesions in which OCT might be particularly useful for assessing and guiding treatment. All patients from ILUMIEN IV with a single target lesion were stratified based on the presence of angiographically moderate or severe calcification, as assessed by the core laboratory, independent of the qualifying characteristics used as inclusion criteria. Moderately and severely calcified lesions were grouped together as differentiating these morphologies may be difficult for the operator in clinical practice. The results of the present analysis suggest that OCT-guided PCI may be more beneficial in patients with angiographically moderately or severely calcified lesions compared with mildly calcified or non-calcified lesions. In concert with the findings from the ILUMIEN IV complex lesion substudy,^13^ which demonstrated improved clinical outcomes with OCT-guided PCI despite the overall neutral results of the main trial that included patients with diabetes even in the absence of complex lesion features, the present analysis supports the hypothesis that the benefit of OCT-guided stent implantation may be more closely related to lesion-level anatomical complexity rather than patient-level clinical characteristics. Our results not only confirm improved procedural and long-term clinical outcomes after OCT-guided PCI compared with angiography-guided PCI in patients with moderately or severely calcified coronary lesions but also suggest potential mechanisms for these benefits. Despite similar pre-PCI angiographic parameters, including the presence of severe calcification, diameter stenosis, and reference vessel diameter, operators more frequently performed advanced lesion preparation using specialty balloons, atherectomy, or intravascular lithotripsy, when guided by OCT rather than angiography alone. Furthermore, they also chose larger stents and devices with OCT guidance, despite knowing that the lesions were severely calcified. After stent implantation, more frequent post-dilatation with higher inflation pressures was performed in the OCT-guided group compared with the angiography-guided group. All of these factors led to greater plaque modification, visible as calcium fractures, a key mechanism of stent expansion in calcified lesions.^6,19,22^ The net effect is that these OCT-directed changes in technique led to a larger final MSA in the OCT-guided group than in the angiography-guided group. However, the magnitude of MSA difference with OCT guidance compared with angiography guidance in this subgroup was slightly less pronounced than what was observed in the overall ILUMIEN IV population.^10^ This finding highlights the inherent challenges in optimizing stent expansion in heavily calcified coronary lesions, which are significantly less compliant than those with none or mild calcification and more prone to procedural complications. Nonetheless, despite more aggressive efforts to achieve greater stent expansion, adverse OCT findings, such as major edge dissection, and peri-procedural MI were less frequently observed in the OCT-guided group compared with the angiography-guided group, suggesting that visualization by intravascular imaging allowed either avoidance or correction of these complications. The net benefit of OCT-guided stent implantation in angiographically moderately or severely calcified lesions thus reflects a combination of enhanced procedural efficacy and safety, which translated into reduced 30-day rates of serious MACE as well as reduced 2-year rates of TVF by 38%, TV-MI by 64%, stent thrombosis by 89%, and serious MACE by 51% in the OCT-guided group compared with the angiography-guided group.

Our study has limitations. First, this was a *post hoc* analysis from the ILUMIEN IV trial, which was not pre-specified to compare primary endpoints in the calcified lesion subgroup. Nonetheless, 2-year TVF rates were higher after PCI with angiographic guidance of lesions with moderate or severe calcification compared with no or mild calcification, and a significant interaction was present between lesion calcification and guidance modality such that OCT guidance reduced 2-year TVF rates in moderately or severely calcified lesions but not in mildly or non-calcified lesions. Second, since randomization was not stratified based on the presence of calcified lesions, there were some mild imbalances between the OCT-guided and angiography-guided groups, which could have affected the outcomes. Nevertheless, the results were robust after adjustment for numerous covariates. Third, pre-PCI OCT was not performed in the angiography-guided group, which limited the detailed assessment of calcified plaque morphology prior to the intervention. Fourth, although a pre-specified PCI protocol was utilized in the OCT-guided group, it did not specify whether or which advanced lesion preparation techniques should be used prior to DES implantation for severely calcified lesions, leaving this to the operators' discretion. Thus, the effectiveness of the OCT-derived calcium score-based advanced lesion preparation strategy^6^ could not be evaluated in the present study. Additionally, we were unable to assess the impact of various advanced lesion preparation strategies on outcomes or compare their relative effectiveness. Fifth, the reduction in TV-MI was driven by reduced peri-procedural MI, the clinical impact of which varies depending on the extent of myonecrosis, which was not collected. Sixth, the study included only patients from ILUMIEN IV with a single target lesion (85% of the entire study population) to minimize potential confounding from events that could arise from other target lesions treated for different qualifying characteristics. Therefore, the findings may not be generalizable to multi-vessel PCI, although we would expect the lessons from the single lesion cohort to apply. Seventh, operators in ILUMIEN IV were experienced in OCT-guided PCI and underwent mandatory training, ensuring a high level of procedural expertise, although this may limit generalizability to less experienced operators. Finally, while the present study did not identify clinical efficacy of OCT guidance in lesions with no/mild calcification, intravascular imaging guidance has been shown to improve clinical outcomes in other complex lesions including long lesions, bifurcations and CTO. Further study is warranted to determine the utility of intravascular imaging guidance in specific complex lesion subgroups without angiographic calcification.

Notwithstanding these limitations, the present report, to the best of our knowledge, is the first substudy of a large-scale RCT that demonstrates a substantial benefit of OCT-guided PCI compared with angiography-guided PCI specifically in patients with angiographically moderately or severely calcified lesions. These results, along with a recent meta-analysis,^12^ provide valuable evidence to guide clinical practice and inform future guidelines, as the most recent European Society of Cardiology guidelines recommend intravascular imaging as a Class IA indication for anatomically complex lesions, such as left main stem, true bifurcations and long lesions, but do not specifically address calcified lesions.^23^

## Conclusions

In patients with angiographically moderately or severely calcified lesions undergoing PCI in the large-scale ILUMIEN IV trial, OCT guidance led to a larger final MSA and reduced the 2-year risks of TVF, a composite of cardiac death, TV-MI, or ID-TVR, compared with angiography guidance, as well as stent thrombosis and both early and late serious MACE. PCI with OCT guidance may be especially beneficial when treating patients with moderate or severe lesion calcification, mitigating the poor prognosis in this high-risk cohort compared with PCI in patients without calcified coronary lesions.

## Supplementary Material

ehaf331_Supplementary_Data

## Declarations

### Data Availability

The data underlying this article are subject to restrictions and cannot be shared publicly.

### Pre-registered Clinical Trial Number

The pre-registered clinical trial number is NCT03507777.

## References

[1] Généreux P, Madhavan MV, Mintz GS, Maehara A, Palmerini T, Lasalle L, et al Ischemic outcomes after coronary intervention of calcified vessels in acute coronary syndromes. Pooled analysis from the HORIZONS-AMI (Harmonizing Outcomes With Revascularization and Stents in Acute Myocardial Infarction) and ACUITY (Acute Catheterization and Urgent Intervention Triage Strategy) TRIALS. J Am Coll Cardiol 2014;63:1845–54. 10.1016/j.jacc.2014.01.034 24561145

[2] Bourantas CV, Zhang YJ, Garg S, Iqbal J, Valgimigli M, Windecker S, et al Prognostic implications of coronary calcification in patients with obstructive coronary artery disease treated by percutaneous coronary intervention: a patient-level pooled analysis of 7 contemporary stent trials. Heart 2014;100:1158–64. 10.1136/heartjnl-2013-305180 24846971

[3] Généreux P, Redfors B, Witzenbichler B, Arsenault MP, Weisz G, Stuckey TD, et al Two-year outcomes after percutaneous coronary intervention of calcified lesions with drug-eluting stents. Int J Cardiol 2017;231:61–7. 10.1016/j.ijcard.2016.12.150 28040289

[4] Barbato E, Gallinoro E, Abdel-Wahab M, Andreini D, Carrié D, Di Mario C, et al Management strategies for heavily calcified coronary stenoses: an EAPCI clinical consensus statement in collaboration with the EURO4C-PCR group. Eur Heart J 2023;44:4340–56. 10.1093/eurheartj/ehad342 37208199

[5] Ali ZA, Shin D, Barbato E. Between a rock and a hard place: a consensus statement on the management of calcified coronary lesions. J Soc Cardiovasc Angiogr Interv 2024;3:101265. 10.1016/j.jscai.2023.101265 39132223 PMC11308428

[6] Fujino A, Mintz GS, Matsumura M, Lee T, Kim SY, Hoshino M, et al A new optical coherence tomography-based calcium scoring system to predict stent underexpansion. EuroIntervention 2018;13:e2182–9. 10.4244/eij-d-17-00962 29400655

[7] Fujii K, Carlier SF, Mintz GS, Mintz GF, Yang Y-M, Yang YF, et al Stent underexpansion and residual reference segment stenosis are related to stent thrombosis after sirolimus-eluting stent implantation: an intravascular ultrasound study. J Am Coll Cardiol 2005;45:995–8. 10.1016/j.jacc.2004.12.066 15808753

[8] Doi H, Maehara AF, Mintz GS, Mintz GF, Yu A, Yu AF, et al Impact of post-intervention minimal stent area on 9–month follow-up patency of paclitaxel-eluting stents: an integrated intravascular ultrasound analysis from the TAXUS IV, V, and VI and TAXUS ATLAS Workhorse, Long Lesion, and Direct Stent Trials. JACC Cardiovasc Interv 2009;2:1269–75. 10.1016/j.jcin.2009.10.005 20129555

[9] Lee JM, Choi KH, Song YB, Lee JY, Lee SJ, Lee SY, et al Intravascular imaging-guided or angiography-guided complex PCI. N Engl J Med 2023;388:1668–79. 10.1056/NEJMoa2216607 36876735

[10] Ali ZA, Landmesser U, Maehara A, Matsumura M, Shlofmitz RA, Guagliumi G, et al Optical coherence tomography-guided versus angiography-guided PCI. N Engl J Med 2023;389:1466–76. 10.1056/NEJMoa2305861 37634188

[11] Holm NR, Andreasen LN, Neghabat O, Laanmets P, Kumsars I, Bennett J, et al OCT or angiography guidance for PCI in complex bifurcation lesions. N Engl J Med 2023;389:1477–87. 10.1056/NEJMoa2307770 37634149

[12] Shin D, Hong D, Singh M, Lee SH, Sakai K, Dakroub A, et al Intravascular imaging-guided percutaneous coronary intervention for heavily calcified coronary lesions: a systematic review and meta-analysis. Int J Cardiovasc Imaging 2024;40:1653–9. 10.1007/s10554-024-03150-7 38874673

[13] Ali ZA, Landmesser U, Maehara A, Shin D, Sakai K, Matsumura M, et al OCT-guided vs angiography-guided coronary stent implantation in complex lesions: an ILUMIEN IV substudy. J Am Coll Cardiol 2024;84:368–78. 10.1016/j.jacc.2024.04.037 38759907

[14] Ali Z, Landmesser U, Karimi Galougahi K, Maehara A, Matsumura M, Shlofmitz RA, et al Optical coherence tomography-guided coronary stent implantation compared to angiography: a multicentre randomised trial in PCI—design and rationale of ILUMIEN IV: OPTIMAL PCI. EuroIntervention 2021;16:1092–9. 10.4244/eij-d-20-00501 32863246 PMC9725042

[15] Madhavan MV, Tarigopula M, Mintz GS, Maehara A, Stone GW, Généreux P. Coronary artery calcification: pathogenesis and prognostic implications. J Am Coll Cardiol 2014;63:1703–14. 10.1016/j.jacc.2014.01.017 24530667

[16] Cutlip DE, Windecker S, Mehran R, Boam A, Cohen DJ, van Es GA, et al Clinical end points in coronary stent trials: a case for standardized definitions. Circulation 2007;115:2344–51. 10.1161/CIRCULATIONAHA.106.685313 17470709

[17] Thygesen K, Alpert JS, Jaffe AS, Chaitman BR, Bax JJ, Morrow DA, et al Fourth universal definition of myocardial infarction (2018). Circulation 2018;138:e618–51. 10.1161/cir.0000000000000617 30571511

[18] Erlinge D, Maehara A, Ben-Yehuda O, Bøtker HE, Maeng M, Kjøller-Hansen L, et al Identification of vulnerable plaques and patients by intracoronary near-infrared spectroscopy and ultrasound (PROSPECT II): a prospective natural history study. Lancet 2021;397:985–95. 10.1016/s0140-6736(21)00249-x 33714389

[19] Zhang M, Matsumura M, Usui E, Noguchi M, Fujimura T, Fall KN, et al Intravascular ultrasound-derived calcium score to predict stent expansion in severely calcified lesions. Circ Cardiovasc Interv 2021;14:e010296. 10.1161/circinterventions.120.010296 34665658

[20] Räber L, Mintz GS, Koskinas KC, Johnson TW, Holm NR, Onuma Y, et al Clinical use of intracoronary imaging. Part 1: guidance and optimization of coronary interventions. An expert consensus document of the European Association of Percutaneous Cardiovascular Interventions. Eur Heart J 2018;39:3281–300. 10.1093/eurheartj/ehy285 29790954

[21] Riley RF, Patel MP, Abbott JD, Bangalore S, Brilakis ES, Croce KJ, et al SCAI expert consensus statement on the management of calcified coronary lesions. J Soc Cardiovasc Angiogr Interv 2024;3:101259. 10.1016/j.jscai.2023.101259 39132214 PMC11307856

[22] Fujino A, Mintz GS, Lee T, Hoshino M, Usui E, Kanaji Y, et al Predictors of calcium fracture derived from balloon angioplasty and its effect on stent expansion assessed by optical coherence tomography. JACC Cardiovasc Interv 2018;11:1015–7. 10.1016/j.jcin.2018.02.004 29798768

[23] Vrints C, Andreotti F, Koskinas KC, Rossello X, Adamo M, Ainslie J, et al 2024 ESC guidelines for the management of chronic coronary syndromes. Eur Heart J 2024;45:3415–537. 10.1093/eurheartj/ehae177 39210710

