# Supplementary material for: Optical coherence tomography- vs angiography-guided coronary stent implantation in calcified lesions: the ILUMIEN IV trial
Source: Eur Heart J. 2025 Jun 5;46(32):3201–10. doi: 10.1093/eurheartj/ehaf331 (PMC12369818; doi:10.1093/eurheartj/ehaf331)
Supplement: ehaf331_Supplementary_Data [file ehaf331_supplementary_data.docx]

# ­Supplementary Appendix

Supplement to: OCT-Guided versus Angiography-Guided Coronary Stent Implantation in Angiographically Calcified Lesions: An ILUMIEN IV Substudy

**Table of Contents**

|  | | Page |
| --- | --- | --- |
| Supplemental Methods 1. | Study Inclusion and Exclusion Criteria | 2-5 |
| Supplemental Methods 2. | Definitions of Procedural Outcomes | 6-8 |
| Supplemental Methods 3. | Definitions of Clinical Outcomes | 9-11 |
| Supplemental Figure 1. | Study Flow | 12 |
| Supplemental Figure 2. | Representative OCT Examples of Complete and Partial Calcium Fracture | 13 |
| Supplemental Figure 3. | Kaplan-Meier Curves of Time to Secondary Clinical Outcomes by Randomization in Patients with Moderately or Severely Calcified Lesions | 14 |
| Supplemental Figure 4. | Subgroup Analysis for 2-year TVF by Randomization in Patients with Moderately or Severely Calcified Lesions | 15 |
| Supplemental Table 1. | Baseline Characteristics of Patients with None or Mildly Calcified Lesions | 16-17 |
| Supplemental Table 2. | Pre-PCI OCT Characteristics of Angiographically Moderately or Severely Calcified Lesions | 18 |

**Supplementary Methods 1. Study Inclusion and Exclusion Criteria**

**1-1. Inclusion Criteria (all must be present)**

1. Subject must be at least 18 years of age.
2. Subject must have evidence of myocardial ischemia (e.g., stable angina, silent ischemia (ischemia in the absence of chest pain or other anginal equivalents), unstable angina, or acute myocardial infarction) suitable for elective PCI.
3. Patients undergoing planned XIENCE stent implantation during a clinically indicated PCI procedure in the ILUMIEN IV trial who were found to have at least one of the following complex angiographic lesion(s);
4. long or multiple lesions (defined as intended total stent length in any single target vessel ≥28 mm),

*Note: For a long target lesion, this would permit treatment by a single long stent or overlapping stents.*

*Note: For up to two target lesions located in a single target vessel and treated with non-overlapping stents, they may be located in a continuous vessel or split up between a main vessel and a side branch.*

1. bifurcation intended to be treated with 2 planned stents (i.e. in both the main branch and side branch), and where the planned side branch stent is ≥ 2.5 mm in diameter by angiographic visual estimation.
2. angiographic severe calcification (defined as angiographically visible calcification on both sides of the vessel wall in the absence of cardiac motion),
3. chronic total occlusion (CTO) (enrolment and randomization in this case performed only after successful antegrade wire escalation crossing and pre-dilatation)
4. in-stent restenosis of diffuse or multi-focal pattern. Lesion must be at or within the existing stent margin(s) and have angiographically visually-assessed DS ≥70% or DS ≥50% with non-invasive or invasive evidence of ischemia
5. All target lesions (those lesions to be randomized) must have a visually estimated or quantitatively assessed %DS of either ≥70%, or ≥50% plus an abnormal functional test (e.g. fractional flow reserve, stress test) signifying ischemia in the distribution of the target lesion(s).
6. All target lesions must be planned for treatment with only ≥2.5 mm and ≤3.5 mm stents and post-dilatation balloons based on pre-PCI angiographic visual estimation.
7. No more than 2 target lesions requiring PCI are present in any single vessel, and no more than 2 target vessels are allowed. Thus, up to 4 randomized target lesions per patient in a maximum of 2 target vessels are allowed, including branches. The intended target lesions will be declared just prior to randomization.

*Note: A lesion is defined as any segment(s) of the coronary tree, no matter how long, which is planned to be covered with one contiguous length of stent, whether single or overlapped. A bifurcation counts as a single lesion even if the side branch is planned to be treated.*

*Note: All lesions in a randomized target vessel that are intended to be treated by PCI are designated as target lesions, and at least one target lesion in each randomized target vessel must meet angiographic high-risk inclusion criteria summarized above in 3B). The only exception is for patients who qualify for the trial on the basis of medication-treated diabetes, in which case no target lesion is required to meet angiographic high-risk inclusion criteria.*

1. All target lesions intended to be treated by PCI in the target vessel are amenable to OCT-guided PCI (i.e. no lesion-specific angiographic exclusion criteria are present).

*Example: If a qualifying angiographic high-risk lesion is in the proximal LAD, and there is a second target lesion in the distal LAD which is a focal lesion not otherwise meeting high-risk criteria, both the proximal LAD and distal LAD lesions must be amenable to OCT (e.g. no excessive tortuosity or calcification precluding delivering the OCT catheter), and each lesion must undergo OCT-guided stenting. Otherwise, the vessel should be excluded from randomization.*

1. Subject must provide written Informed Consent prior to any study related procedure.

**1-2. Exclusion Criteria (none may be present)**

1. **Clinical exclusion criteria:**
2. STEMI ≤24 hours from the onset of ischemic symptoms
3. Creatinine clearance ≤30 ml/min/1.73 m^2^ (as calculated by MDRD formula for estimated GFR)^[[1]](#footnote-2)^ and not on dialysis. Note: chronic dialysis dependent patients are eligible for enrolment regardless of creatinine clearance.
4. Hypotension, shock or need for mechanical support or intravenous vasopressors at the time the patient would be undergoing the index procedure.
5. CHF (Killip class ≥2 or NYHA class ≥3)
6. LVEF ≤30% by the most recent imaging test within 3 months prior to procedure. If no LVEF test result within 3 months is available, it must be assessed by echocardiography, multiple gated acquisition (MUGA), magnetic resonance imaging (MRI), ventriculography (LV gram) or other method.
7. Unstable ventricular arrhythmias
8. Inability to take DAPT (both aspirin and a P2Y12 inhibitor) for at least 12 months in the patient presenting with an ACS, or at least 6 months in the patient presenting with stable CAD, unless the patient is also taking chronic oral anticoagulation in which case a shorter duration of DAPT may be prescribed per local standard of care.
9. Planned major cardiac or non-cardiac surgery within 24 months after the index procedure
10. *Note: Major surgery is any invasive operative procedure in which an extensive resection is performed, e.g. a body cavity is entered, organs are removed, or normal anatomy is altered.*
11. *Note: Minor surgery is an operation on the superficial structures of the body or a manipulative procedure that does not involve a serious risk. Planned minor surgery is not excluded.*
12. Prior PCI within the target vessel within 12 months
13. *Note: Prior PCI within the target vessel within 12 months is allowed for in-stent restenosis (target lesion is the prior PCI site) if no more than one layer of previously implanted stent is present.*
14. *Note: In-stent restenosis involving two or more layers of stent implanted at any time prior to index procedure (i.e. an earlier episode of in-stent restenosis previously treated with a second stent) is excluded.*
15. Any planned PCI within the target vessel(s) within 24 months after the study procedure, other than a planned staged intervention in a second randomized target vessel.
16. *Note: Planned staged interventions must be noted at the time of randomization, and the decision to stage may be modified within 24 hours of completion of the index PCI. See* ***Section*** ***6.5.3.8*** *for more details of multi lesion and vessel treatment.*
17. *Note: PCI in non-target vessels is permitted >48 hours after the index procedure.*
18. Any prior PCI in a non-target vessel within 24 hours before the study procedure, or within previous 30 days if unsuccessful or complicated.
19. *Note: Patients requiring non-target vessel PCI may be enrolled and the non-target vessel(s) may be treated in the same index procedure as the randomized lesions (in all cases prior to randomization), as long as treatment of the lesion(s) in the non-target vessel is successful and uncomplicated.*
20. *Successful and uncomplicated* definition for non-target vessel treatment during the index procedure: Angiographic diameter stenosis <10% for all treated non-target lesions, with TIMI III flow in this vessel, without final dissection ≥ NHLBI type B, perforation anytime during the procedure, prolonged chest pain (>5 minutes) or prolonged ST-segment elevation or depression (>5 minutes), or cardiac arrest or need for defibrillation or cardioversion or hypotension/heart failure requiring mechanical or intravenous hemodynamic support or intubation).
21. Subject has known hypersensitivity or contraindication to any of the study drugs (including all P2Y12 inhibitors, one or more components of the study devices, including everolimus, cobalt, chromium, nickel, platinum, tungsten, acrylic and fluoropolymers, or radiocontrast dye that cannot be adequately pre-medicated.
22. Subject has received a solid organ transplant which is functioning or is active on a waiting list for any solid organ transplants with expected transplantation within 24 months.
23. Subject is receiving immunosuppressant therapy or has known immunosuppressive or severe autoimmune disease that requires chronic immunosuppressive therapy (e.g., human immunodeficiency virus, systemic lupus erythematosus, etc.). Note: corticosteroids are not included as immunosuppressant therapy.
24. Subject has previously received or is scheduled to receive radiotherapy to a coronary artery (vascular brachytherapy), or the chest/mediastinum.
25. Subject has a platelet count <100,000 cells/mm^3^ or >700,000 cells/mm^3^.
26. Subject has a documented or suspected hepatic disorder as defined as cirrhosis or Child-Pugh ≥ Class B.
27. Subject has a history of bleeding diathesis or coagulopathy, or has had a significant gastro-intestinal or significant urinary bleed within the past six months.
28. Subject has had a cerebrovascular accident or transient ischemic neurological attack (TIA) within the past six months, or any prior intracranial bleed, or any permanent neurologic defect, or any known intracranial pathology (e.g., aneurysm, arteriovenous malformation, etc.).
29. Subject has extensive peripheral vascular disease that precludes safe 6 French sheath insertion. Note: femoral arterial disease does not exclude the patient if radial access may be used.
30. Subject has life expectancy <2 years for any non-cardiac cause.
31. Subject is currently participating in another investigational drug or device clinical study that has not yet completed its primary endpoint^[[2]](#footnote-3)^.
32. Pregnant or nursing subjects and those who plan pregnancy in the period up to 2 years following index procedure. Female subjects of child-bearing potential must have a negative pregnancy test done within 7 days prior to the index procedure per site standard test.
33. Presence of other anatomic or comorbid conditions, or other medical, social, or psychological conditions that, in the investigator’s opinion, could limit the subject’s ability to participate in the clinical investigation or to comply with follow-up requirements, or impact the scientific soundness of the clinical investigation results.
34. **Angiographic exclusion criteria:**
35. Syntax score ≥33, unless a formal meeting of the Heart Team, including a cardiac surgeon, concludes that PCI is appropriate.
36. Planned use of any stent <2.5 mm in a target vessel based on visual estimation (note: a smaller stent may be used in a bail-out scenario – e.g. to treat a distal dissection – but its use cannot be planned prior to enrolment)
37. Planned use of a stent or post-dilatation balloon ≥3.75 mm for the target lesion (see inclusion criteria #5 for the one exception to this exclusion criterion)
38. Severe vessel tortuosity or calcification in a target vessel such that it is unlikely that the OCT catheter can be delivered (note: severe vessel calcification is allowed if it is expected that the OCT catheter can be delivered at baseline or after vessel preparation with balloon pre-dilatation or atherectomy)
39. The target vessel has a lesion with DS ≥ 50% that is not planned for treatment at the time of index procedure.
40. The target lesion is in the left main coronary artery
41. The target lesion is in a bypass graft conduit. Note: A native coronary artery may be randomized if a prior bypass graft conduit to the vessel is totally occluded, but not if it is patent.
42. The target lesion is an ostial RCA stenosis
43. The target lesion is a stent thrombosis
44. Planned use of any stent other than Xience in a target lesion

**Supplemental Methods 2. Definitions of Procedural Outcomes**

**2-1. OCT parameters (core laboratory assessed, analyzed per target lesion)**

- 1. Stent expansion. Stent expansion is defined by the MSA achieved in the proximal and distal stented segments relative to their respective reference lumen areas. The stent length is divided into 2 equal segments (proximal and distal) except for lesions containing a bifurcation (visually estimated side branch ≥2.5 mm). When there is a bifurcation present, rather than splitting the stent into two halves, the division occurs at the proximal most side branch.
- Acceptable stent expansion (categorical variable): The MSA of the proximal segment is ≥90% of the proximal reference lumen area and the MSA of the distal segment is ≥90% of the distal reference lumen area.
- Unacceptable stent expansion (categorical variable): The MSA of the proximal segment is <90% of the proximal reference lumen area, and/or the MSA of the distal segment is <90% of the distal reference lumen area.

In case either segment (proximal or distal) of the stent meets criteria for unacceptable stent expansion, the stent is considered to have unacceptable stent expansion. Both segments of the stent must meet acceptable stent expansion criteria to be considered acceptable.

In case a respective reference segment cannot be measured the determination will be made with only one of the two reference (proximal or distal) segments

*Note: If acceptable stent expansion (by operator assessment) is not achieved in either the distal or proximal segments of the stent in the OCT-guided arm according to the Post-PCI OCT, further post-stent expansion with higher pressures and/or larger balloons must be performed per protocol if the POST-PCI OCT EEL measurements now suggest a larger balloon be used.*

- Post-PCI stent expansion (%) (continuous variable): The MSA divided by the average of proximal and distal reference lumen areas x 100

1. Mean stent expansion (%) (continuous variable): The mean stent area (stent volume/analysed stent length) divided by the average of proximal and distal reference lumen areas x 100.
2. Intra-stent plaque protrusion and thrombus. Defined as a mass attached to the luminal surface or floating within the lumen, meeting the following criteria: Protrusion/thrombus is defined as any intraluminal mass protruding at least 0.2 mm within the luminal edge of a stent strut, and will be further classified as Major and Minor:

- Major: Protrusion area/Stent area at site of tissue protrusion ≥10% and the minimal intrastent flow area (MSA – protrusion area) is unacceptable (<90% of respective proximal or distal reference area
- Minor: Protrusion area/Stent area at site of tissue protrusion is <10%, or is ≥10% but the minimal intraluminal flow area (MSA – protrusion area) is acceptable (≥90% of respective proximal or distal reference area

Note: It is recommended that if protrusion is detected by operator assessment in the OCT-guided arm during the procedure and meets the criteria for major protrusion, then thrombus aspiration, further high-pressure balloon inflation and/or an additional stent be considered.

1. Untreated reference segment disease. Defined as focal disease with untreated MLA <4.5 mm^2^ within 5 mm from the proximal and/or distal stent edges. Sub-classified by the amount of untreated lipid plaque, divided into 3 grades:

- Low (≤90° of lipid arc)
- Medium (>90°-<180° of lipid arc)
- High (≥180° of lipid arc)

*Note: If untreated reference segment disease with an MLA <4.5 mm^2^ is detected by operator assessment in either the proximal reference (inflow disease) or distal reference (outflow disease) segment lumen in the OCT-guided arm, an additional stent must be placed to treat it, unless there are anatomic reasons that the disease should not be covered (e.g. diffuse distal disease or significant vessel tapering, etc.) (Figures 1a-b).*

1. Edge dissections. Edge dissections will be tabulated as:

- Major (%): ≥60 degrees of the circumference of the vessel at site of dissection and ≥3 mm in length
- Minor (%): any visible edge dissection <60 degrees of the circumference of the vessel or <3 mm in length

1. Stent Malapposition. Defined as frequency (%) of incompletely apposed stent struts (defined as stent struts clearly separated from the vessel wall (lumen border/plaque surface) without any tissue behind the struts with a distance from the adjacent intima of ≥0.2 mm and not associated with any side branch).

Malapposition will be further classified as:

- Major: if associated with unacceptable stent expansion (as defined above)
- Minor: if associated with acceptable stent expansion (as defined above)

Note: If malapposition is detected by operator assessment during the procedure in the OCT-guided arm and meets the criteria for major malapposition (i.e. malapposition associated with unacceptable stent expansion), further stent expansion must be performed. The degree of stent underexpansion (acceptable or unacceptable) should guide the intervention rather than amount of malapposition.

1. Border detection (angiography arm post-PCI only, blinded to investigator)

The visibility of the vessel external elastic lamina (EEL) border by OCT will be evaluated at both reference sites (proximal and distal) and the MSA, after intervention and then classified into 3 grades:

- Good: ≥75% (270°) of visible circumference
- Moderate: ≥50% (180°) - <75% (270°) of visible circumference
- Poor: <50% (180°) of visible circumference

1. Intra-stent lumen area (intra-stent flow area). Defined as stent area minus any protrusion as defined above in secondary endpoint 3) (Intra-stent plaque protrusion and thrombus).
2. Effective lumen area (total flow area). Defined as intra-stent lumen area plus any area of malapposition between the stent and the vessel wall (lumen border/plaque border).

**2-2. Angiographic outcomes (core laboratory assessed; analyzed per target lesion**)

1. Reference Vessel Diameter (RVD): Average diameter of proximal and distal healthy segments by QCA. “Normal” reference segments are selected proximal and distal to the stenosis and averaged to define the reference vessel diameter.
2. Final (post-PCI) minimal lumen diameter: The smallest measured luminal diameter in a diseased segment (as measured by QCA).
3. Final (post-PCI) percent diameter stenosis: The value calculated as 100 * (1 - MLD/RVD) using the mean values from two orthogonal views (when possible) by QCA.
4. Acute lumen gain: The difference between the post-PCI MLD and the pre-PCI MLD (as measured by QCA).
5. Post-PCI target vessel TIMI flow rate (vessel level analysis)
6. Angiographic complications – worst (anytime during the procedure) and final (post PCI and all imaging) - Angiographic dissection ≥ NHLBI type B, perforations (Ellis classification), intra-procedural thrombotic events (including slow-flow, no-reflow, side branch closure, distal embolization, and intra-procedural stent thrombosis, as per the standard angiographic core laboratory definitions

**Supplemental Methods 3. Definitions of Clinical Outcomes**

1. **Death (Per ARC Circulation 2007; 115: 2344-2351):** All deaths are considered cardiac unless an unequivocal non-cardiac cause can be established. Specifically, any unexpected death even in patients with coexisting potentially fatal non-cardiac disease (e.g. cancer, infection) should be classified as cardiac.

- **Cardiac death:** Any death due to proximate cardiac cause (e.g. MI, low-output failure, fatal arrhythmia), unwitnessed death and death of unknown cause, all procedure related deaths including those related to concomitant treatment.
- **Vascular death:** Death due to non-coronary vascular causes such as cerebrovascular disease, pulmonary embolism, ruptured aortic aneurysm, dissecting aneurysm, or other vascular cause.
- **Non-cardiovascular death:** Any death not covered by the above definitions such as death caused by infection, malignancy, sepsis, pulmonary causes, accident, suicide or trauma.

1. **Myocardial Infarction (Primary Protocol Definition)**
2. **Periprocedural MI (Modified ARC-2 Definition).**

Periprocedural MI occurring within 48 hours after all percutaneous coronary intervention (PCI) and coronary artery bypass grafting (CABG) procedures

Absolute rise (from baseline to within 48 hours of procedure) in cardiac troponin of ≥35x the 99th percentile URL (or ≥35x ULN if URL is not available) or in the absence of cardiac troponin, rise in CK-MB to ≥ 5x the 99th percentile URL (or ≥5x ULN if URL is not available). Note, cardiac troponin assessments are preferentially used if available; otherwise CK-MB may be used.

In addition, 1 (or more) of the following measures of myocardial ischemia must be present post-procedure:

- New significant Q waves (≥40 ms in duration and ≥1 mm deep in voltage in ≥2 contiguous leads) or equivalent
- Persistent flow-limiting angiographic complications in a major epicardial vessel or branch ≥1.5 mm in diameter present at the end of the PCI procedure (or during angiography performed to evaluate a post-CABG complication) as assessed at the angiographic core laboratory
- New substantial loss of viable myocardium on serial imaging

These assessments apply to patients: a) with baseline CK-MB or cardiac troponin levels ≤1x the 99th percentile URL (or ≤1x ULN if URL is not available); b) in whom the baseline biomarker is >1x the 99th percentile URL (or >1x ULN if URL is not available) and stable or falling; and c) with a single elevated baseline draw who have a chronic coronary syndrome (CCS). In the latter two groups (patients in whom the baseline is elevated and stable or falling and CCS patients with a single elevated baseline draw), the post-procedural troponin (or CK-MB) must rise above the most recent baseline by an increment of the values above (i.e. for troponin ≥35x the 99th percentile URL (or ≥35x ULN if URL is not available) or in the absence of cardiac troponin, for CK-MB ≥5x the 99th percentile URL (or ≥5x ULN if URL is not available).

These assessments do not apply to patients: a) in whom baseline CK-MB or troponin levels are elevated and rising; and b) with a single elevated baseline level who presented with a NSTEMI or STEMI in whom it is uncertain whether the peak has been reached. In such patients periprocedural MI will only be adjudicated if the troponin (or CK-MB) biomarker increases from the prior measure by the increments above, and at least 2 of the 3 above criteria for myocardial ischemia are present.

1. **Spontaneous MI - 4^th^ Universal Definition of MI classification**

All MIs which are not peri-procedural are considered spontaneous MIs. Spontaneous MIs are defined and subclassified according to types 1, 2, 3, 4b and 4c according to the 4^th^ Universal Definition of MI classification^1^ (below).

1. Type 1: MI caused by atherothrombotic coronary artery disease (CAD) and usually precipitated by atherosclerotic plaque disruption (rupture or erosion) is designated as a Type 1 MI. Type 1 MI is characterized by detection of a rise and/or fall of cTn values with at least one value above the 99th percentile URL and with at least one of the following:

- Symptoms of acute myocardial ischemia;
- New ischemic ECG changes;
- Development of pathological Q waves;
- Imaging evidence of new loss of viable myocardium or new regional wall motion abnormality in a pattern consistent with an ischemic etiology;
- Identification of a coronary thrombus by angiography including intracoronary imaging or by autopsy

1. Type 2: Myocardial infarction secondary to an ischemic imbalance. The pathophysiological mechanism leading to ischemic myocardial injury in the context of a mismatch between oxygen supply and demand has been classified as type 2 MI. Type 2 MI is characterized by detection of a rise and/or fall of cTn values with at least one value above the 99th percentile URL, and evidence of an imbalance between myocardial oxygen supply and demand unrelated to coronary thrombosis, requiring at least one of the following:

- Symptoms of acute myocardial ischemia;
- New ischemic ECG changes;
- Development of pathological Q waves;
- Imaging evidence of new loss of viable myocardium or new regional wall motion abnormality in a pattern consistent with an ischemic etiology

1. Type 3: Myocardial infarction resulting in death when biomarker values are unavailable. Patients are designated as having Type 3 MI who suffer cardiac death, with symptoms suggestive of myocardial ischemia accompanied by presumed new ischemic ECG changes or ventricular fibrillation, but die before blood samples for biomarkers can be obtained, or before increases in cardiac biomarkers can be identified, or MI is detected by autopsy examination.
2. Type 4b: Myocardial infarction related to stent thrombosis associated with percutaneous coronary intervention (PCI). Myocardial infarction associated with stent thrombosis is designated as Type 4b MI, detected by angiography or autopsy using the same criteria utilized for Type 1 MI.
3. Type 4c: Myocardial infarction related to restenosis associated with percutaneous coronary intervention (PCI). This PCI-related MI type is designated as Type 4c MI, defined as focal or diffuse restenosis, or a complex lesion associated with a rise and/or fall of cTn values above the 99th percentile URL applying, the same criteria utilized for Type 1 MI.
4. **Myocardial Infarction Relation to Target Vessel:**

Infarcts will be adjudicated according to anatomical origin based on review of coronary angiography performed at the time of the event. Those that cannot be clearly attributed to a particular vessel (target or non-target) either because the origin of the event on the angiogram is ambiguous or the angiogram was not performed will be considered as indeterminate vessel MI.

1. **Intraprocedural stent thrombosis:** new or increasing thrombus developing within or adjacent to the stent during the index PCI procedure.
2. **Stent Thrombosis (definite or probable; modified ARC definitions):**
   1. **Stent Thrombosis, Definite*:** Definite stent thrombosis is considered to have occurred by either angiographic or pathological confirmation.

Angiographic confirmation of stent thrombosis

The presence of a thrombus that originates in the stent or in the segment 5 mm proximal or distal to the stent AND presence of at least 1 of the following criteria within a 48-hour time window:

- Acute onset of ischemic symptoms at rest
- New ischemic ECG changes that suggest acute ischemia
- Typical rise and fall in cardiac biomarkers (refer to definition of spontaneous MI)

* The incidental angiographic documentation of stent occlusion in the absence of clinical signs or symptoms is not considered a confirmed stent thrombosis (silent occlusion).

- 1. **Stent Thrombosis, Probable:** Clinical definition of probable stent thrombosis is considered to have occurred after intracoronary stenting in the following cases:
- Any unexplained death within the first 30 days.
- Irrespective of the time after the index procedure, any MI that is related to documented acute ischemia in the territory of the implanted stent without angiographic confirmation of stent thrombosis and in the absence of any other obvious cause.

1. **Ischemia-Driven [ID] Revascularization:** A revascularization is considered ischemia driven if associated with any of the following:

- Positive functional ischemia study including positive FFR, iFR, etc.
- Ischemic symptoms and angiographic diameter stenosis ≥50% by core laboratory QCA
- Angiographic diameter stenosis ≥ 70% by core laboratory QCA without angina or positive functional study

**Supplementary Figure 1. Study Flow**

OCT, optical coherence tomography

**
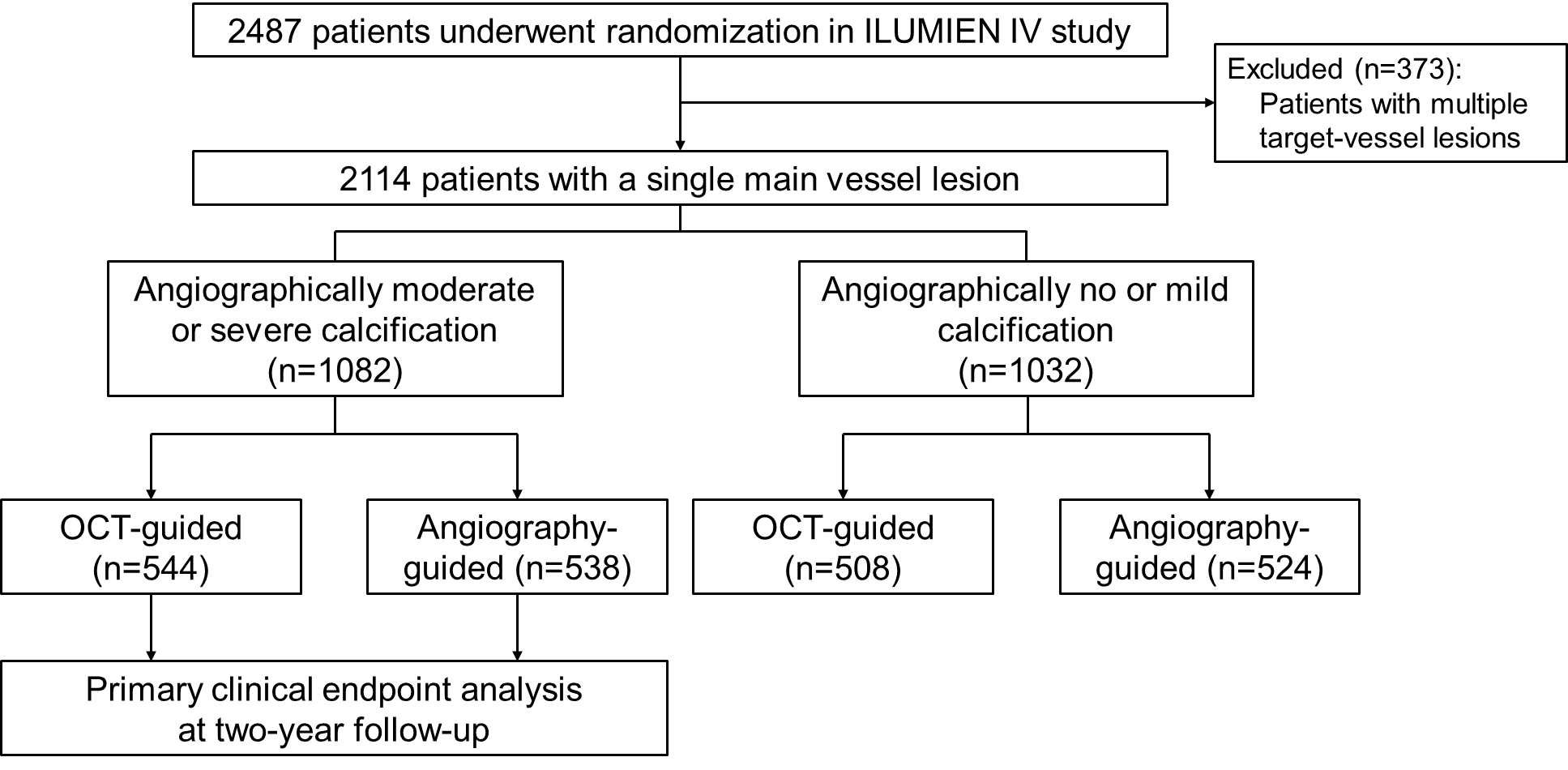
**

**Supplemental Figure 2. Representative OCT Examples of Complete and Partial Calcium Fracture**

Representative examples of (A) complete and (B) partial calcium fractures (white arrows) on OCT are presented. Complete fracture is defined as a new disruption through the full thickness of a calcified plaque. Partial fracture is defined as a new disruption that does not extend through the entire thickness of the calcified plaque.

OCT, optical coherence tomography.


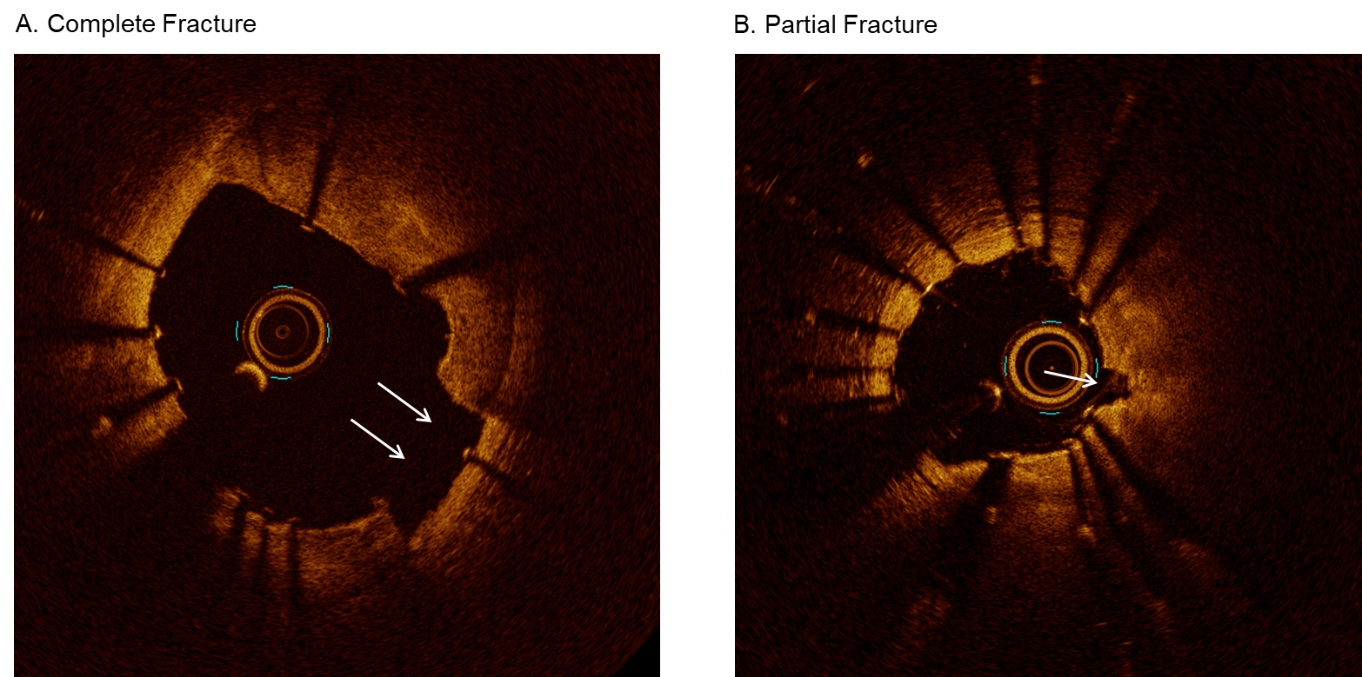


**Supplemental Figure 3. Kaplan-Meier Curves of Time to Secondary Clinical Outcomes by Randomization in Patients with Moderately or Severely Calcified Lesions**

A) Cardiac death, B) TV-MI, C) ID-TVR, and D) definite or probable stent thrombosis.

aHR, adjusted hazard ratio; CI, confidence interval; ID-TVR, ischemia driven-target vessel revascularization; OCT, optical coherence tomography; PCI, percutaneous coronary intervention; TV-MI, target-vessel myocardial infarction.


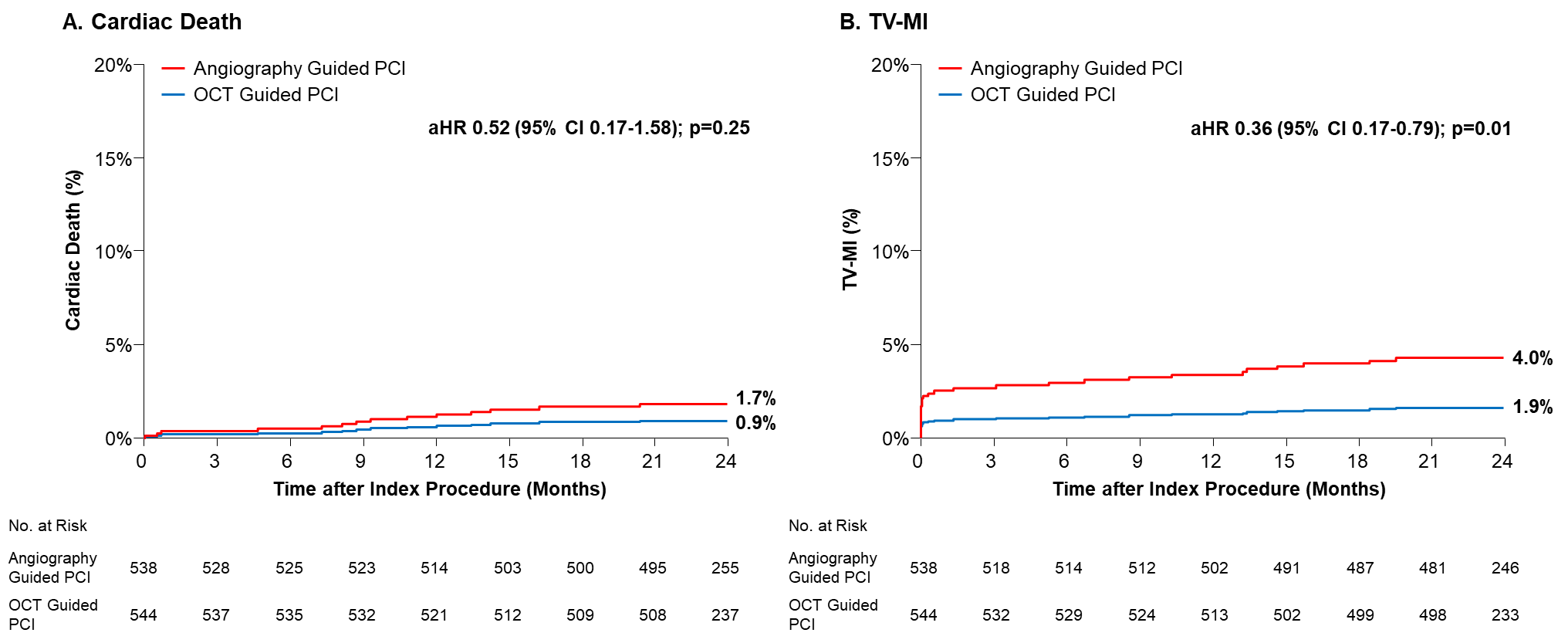


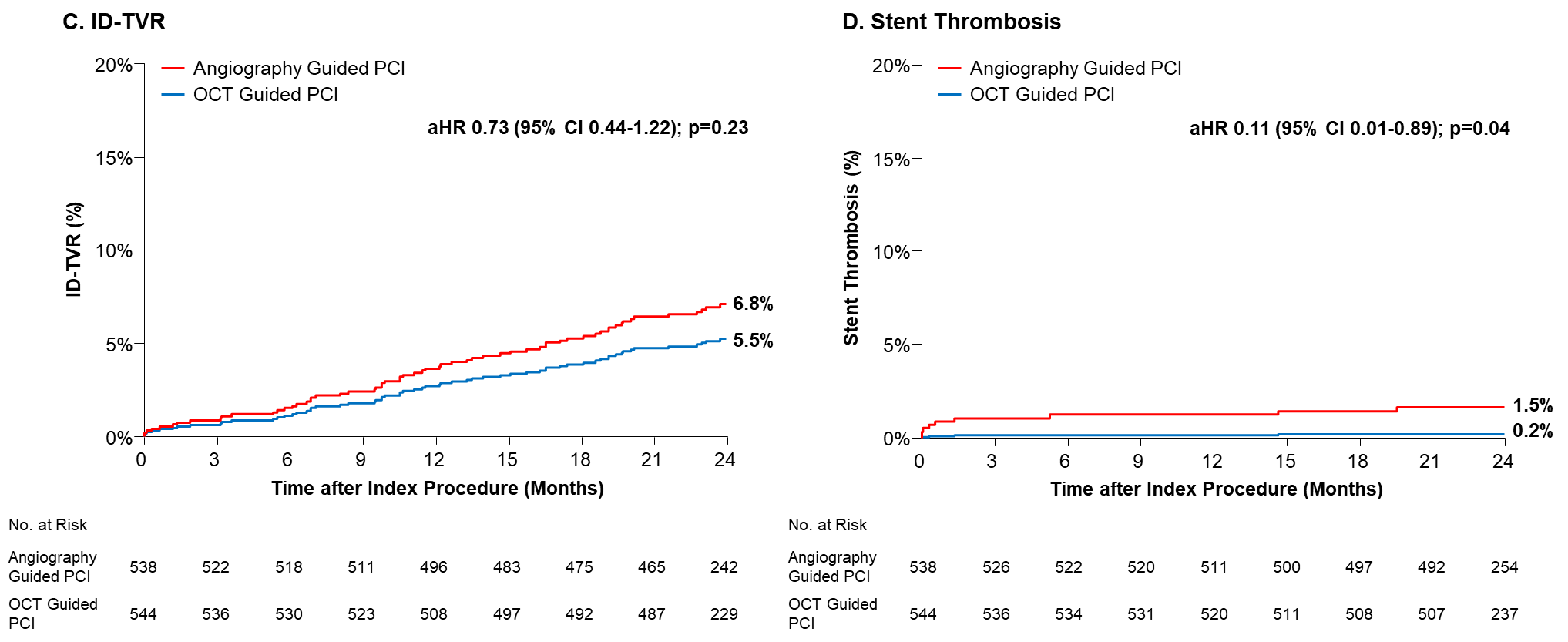


**Supplemental Figure 4. Subgroup Analysis of 2-Year TVF by Randomization in Patients with Moderately or Severely Calcified Lesions**

ACS, acute coronary syndromes; aHR, adjusted hazard ratio; CI, confidence interval; MI, myocardial infarction; NA, not available; OCT, optical coherence tomography; TVF, target-vessel failure.

**
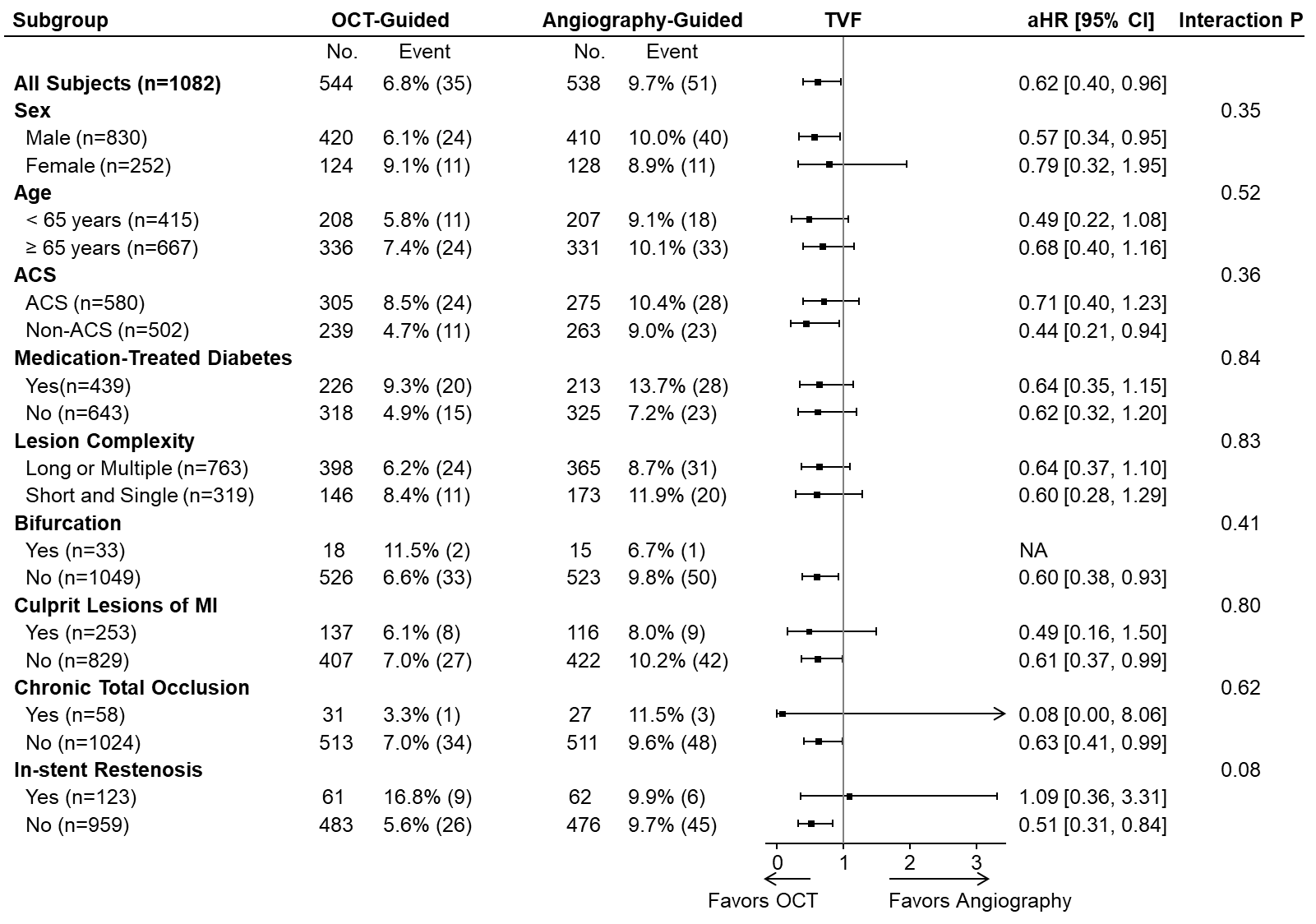
**

**Supplemental Table 1. Baseline Characteristics of Patients with No or Mildly Calcified Lesions**

| **Characteristic** | **OCT-Guided**  **(N=508)** | **Angiography-Guided**  **(N=524)** | **P-value** |
| --- | --- | --- | --- |
| Age, yr | 63.1 ± 10.5 | 63.9 ± 10.5 | 0.19 |
| Male | 405 (79.7%) | 391 (74.6%) | 0.05 |
| BMI, kg/m^2^ | 28.4 ± 4.8 | 28.9 ± 5.4 | 0.17 |
| Hypertension | 348 (68.5%) | 368 (70.2%) | 0.55 |
| Dyslipidemia | 329 (64.8%) | 354 (67.6%) | 0.34 |
| Current or recent smoker | 120 (23.6%) | 130 (24.8%) | 0.66 |
| Peripheral vascular disease | 33 (6.5%) | 32 (6.1%) | 0.80 |
| Prior myocardial infarction | 110 (21.7%) | 136 (26.0%) | 0.11 |
| Prior PCI in the target vessel | 61/501 (12.2%) | 59/520 (11.3%) | 0.68 |
| Prior CABG | 17 (3.3%) | 15 (2.9%) | 0.65 |
| Renal insufficiency | 37 (7.3%) | 40 (7.6%) | 0.83 |
| Dialysis | 8 (1.6%) | 9 (1.7%) | 0.86 |
| Left ventricular ejection fraction, % | 55.1 ± 8.5 | 55.3 ± 8.5 | 0.81 |
| Clinical presentation |  |  |  |
| Silent ischemia | 63 (12.4%) | 75 (14.3%) | 0.37 |
| Stable angina | 137 (27.0%) | 136 (26.0%) | 0.71 |
| Unstable angina | 124 (24.4%) | 130 (24.8%) | 0.88 |
| NSTEMI | 153 (30.1%) | 153 (29.2%) | 0.75 |
| Recent STEMI (>24 hours) | 31 (6.1%) | 30 (5.7%) | 0.80 |
| Qualifying characteristics† |  |  |  |
| Diabetes mellitus, medication-treated | 188/507 (37.1%) | 203/522 (38.9%) | 0.59 |
| Culprit lesion NSTEMI | 150/507 (29.6%) | 153/522 (29.3%) | 0.98 |
| Culprit lesion STEMI >24 hours | 31/507 (6.1%) | 30/522 (5.7%) | 0.91 |
| Long or multiple lesions‡ | 317/507 (62.5%) | 322/522 (61.7%) | 0.83 |
| Two-stent bifurcation§ | 18/507 (3.6%) | 21/522 (4.0%) | 0.82 |
| Severe calcification¶ | 22/507 (4.3%) | 24/522 (4.6%) | 0.96 |
| Chronic total occlusion║ | 42/507 (8.3%) | 35/522 (6.7%) | 0.40 |
| Diffuse or multi-focal ISR | 51/507 (10.1%) | 52/522 (10.0%) | 1.00 |

Data are presented as means ± standard deviation or number (percentage).

* Recent smoking within 30 days

† Patients may have more than one qualifying characteristic.

‡ Total stent length ≥28mm

§ Intended for treatment with a stent ≥2.5 mm in diameter in both the main vessel and side-branch vessel.

¶ Defined as visible calcification on both sides of the vessel wall in the absence of cardiac motion.

║ After successful crossing with antegrade wire escalation and pre-dilatation.

CABG, coronary artery bypass graft; ISR, in-stent restenosis; NSTEMI, non-ST-segment elevation myocardial infarction; PCI, percutaneous coronary intervention; STEMI, ST-segment elevation myocardial infarction.

**Supplemental Table 2. Pre-PCI OCT Characteristics of Angiographically Moderately or Severely Calcified Lesions**

| **Characteristic** | **OCT-Guided Group**  **(L=532)*** |
| --- | --- |
| Minimal lumen area, mm^2^ | 1.95 ± 0.81 |
| Mean reference lumen area, mm^2^ | 6.55 ± 2.08 |
| Area stenosis at MLA site, % | 68.9 ± 12.3 |
| Lesion length, mm | 38.6 ± 15.7 |
| Maximum superficial calcium arc, º | 205.1 ± 99.5 |
| Maximum superficial calcium thickness, mm | 0.82 ± 0.27 |
| Dominant plaque characteristics |  |
| Native atherosclerotic plaque | 476 (89.5%) |
| In-stent restenosis (includes edge restenosis) | 56 (10.5%) |
| Neointimal hyperplasia | 15 (2.8%) |
| Stent underexpansion | 6 (1.1%) |
| Neoatherosclerosis | 35 (6.6%) |

*The total number of main vessel lesions from the OCT core laboratory available for pre-PCI OCT analysis.

MLA, minimal lumen area; OCT, optical coherence tomography; PCI, percutaneous coronary intervention.

1. Estimated GFR (ml/min/ 1.73 m^2^) = 175 x [SerumCreatinine (umol/L) x 0.0113]^-1.154^ x Age (years)^-0.203^  (x 0.742 if female) [↑](#footnote-ref-2)
2. This includes clinical trials of medications and invasive procedures. Questionnaire-based studies, or other studies (regardless of study design) that are non-invasive and do not require medication are allowed. A subject who is taking part in the long-term follow-up phase of a trial, who has completed all medications and invasive procedures per protocol requirements, may continue to participate in that trial. [↑](#footnote-ref-3)
